# Pregnancy Rate Following Luteal Phase Support in Iranian Women with Polycystic Ovarian Syndrome 

**Published:** 2014-11-01

**Authors:** Fatemeh Foroozanfard, Hamidreza Saberi, Seyed Alireza Moraveji, Fatemeh Bazarganipour

**Affiliations:** 1 Gametogenesis Research Center, Kashan University of Medical Sciences, Kashan, Iran; 2 Department of Occupational Health, Kashan University of Medical Sciences, Kashan, Iran; 3 Department of Social Medicine, Kashan University of Medical Sciences, Kashan, Iran; 4 Hormozgan Fertility and Infertility Research Center, Hormozgan University of Medical Sciences, Bandarabbas, Iran

**Keywords:** Clomiphene, Letrozole, Progesterone, Luteal Phase, Polycystic Ovarian Syndrome

## Abstract

**Background:**

To assess the efficacy of luteal phase support (LPS) using intravaginal progesterone (P) on pregnancy rate in Iranian women with polycystic ovarian syndrome (PCOS) who used a combination for ovulation induction consisting of letrozole or clomi- phene citrate (CC) and human menopausal gonadotropin (HMG).

**Materials and Methods:**

This was a randomized clinical trial undertaken in a fertility clinic in Kashan, Isfahan Province, Iran. A total of 198 patients completed treatment and follow up. Base on chosen ovulation induction programs, they were divided into two following group: i. CC group (n=98) used a combination consisting of CC (100 mg×5 day) and HMG (150 IU×5 day) and ii. letrozole group (n=100) used a combination consisting of letrozole (5 mg×5 day) and HMG (150 IU×5 day). After human chorionic gonadotropin (hCG) administration (5000 IU), the patients (n=122) who randomly re- ceived intravaginal P (Cyclogest, 400 mg daily) were included in LPS group, while the rest (n=123) were included in non-P cycles group. The outcome was the comparison of chemical pregnancy rate between the groups.

**Results:**

Our findings showed that LPS was associated with a 10% higher pregnancy rate than in non-P cycles, although this difference did not reach statistical significant (p=0.08). LPS improved pregnancy rate in both CC (4%) and letrozole (6%) groups. In addition, patients who used letrozole for ovulation induction along with intravaginal P showed higher pregnancy rates than CC group.

**Conclusion:**

Administration of vaginal P for LPS may improve the pregnancy rate in women with PCOS using letrozole or CC in combination with HMG for ovulation induc- tion (Registration Number: IRCT201206072967N4).

## Introduction

The luteal phase has been defined as the period between ovulation and either the establishment of a pregnancy or the onset of menses two weeks later. Luteal phase defect (LPD) has been attributed mainly to inadequate production of progesterone (P) that is known as the major product of the corpus luteum, which is necessary for the establishment of pregnancy. As a result, P has been used as luteal phase support (LPS) in ovulation induction cycles for many years (1).

LPD has been reported in patients with polycystic ovarian syndrome (PCOS) that has been identified as most common endocrine disorder in women of reproductive age (2). This type of disorder causing abnormal follicular development and numerous antral follicles may be related to abnormal hypothalamic sensitivity to P. Furthermore, the granulosa cells of women with PCOS may have an inherent inability to secrete normal levels of P after luteinization if ovulation is achieved (3).

On the other hand, controlled ovarian hyperstimulation is generally used as treatment protocols for patients with PCOS. In controlled ovarian hyperstimulation cycles, multifollicular development and supraphysiologic steroid serum concentrations may negatively affect luteinizing hormone (LH) secretion. Disturbed LH secretion may induced LPD that leads to premature luteolysis, reduced LH concentration, low P level and shortened luteal phase (4).

Some studies have been shown that presence of LPS through administration of P has significantly affected the success of ovarian induction and intrauterine insemination (IUI) cycles (5, 6). Nevertheless, in the studies done by Ozornek et al. (7) and Kyrou et al. (8) they reported no benefit of LPS in patients who underwent stimulated IUI cycles. In another study has been concluded that P supplementations have low therapeutic value in LPD, beside taking clomiphene citrate (CC) for ovulation induction (9). Montville et al. strongly recommended luteal phase supplementations containing P in women with PCOS using letrozole for ovulation induction, while no positive effect of P on those stimulated with clomiphene citrate was detected (10).

Therefore, the previous studies have produced conflicting results, while the amount of data from well-controlled clinical trials is limited. Thus, further studies are required to describe the impact of treatment with P for LPS in stimulated cycles in PCOS before deciding to move forward with more invasive assisted reproductive technologies.

To best our knowledge, there had been no prospective trial investigating the need for P administration in the combination stimulation protocols in PCOS. In light of these observations, the aim of present study was to evaluate the effect of LPS with P on pregnancy rate in Iranian women with PCOS who were treated with either CC or letrozole in combination with human menopausal gonadotropin (HMG).

## Materials and Methods

A randomized clinical trial with parallel design was employed to confirm the effect of LPS with P on pregnancy rate in patients with PCOS. This study was conducted in an infertility clinic affiliated with Shahid Beheshti Hospital in Kashan, Isfahan Province, central part of Iran, between Aprils and January 2011.

### Patient population

**Fig 1:**
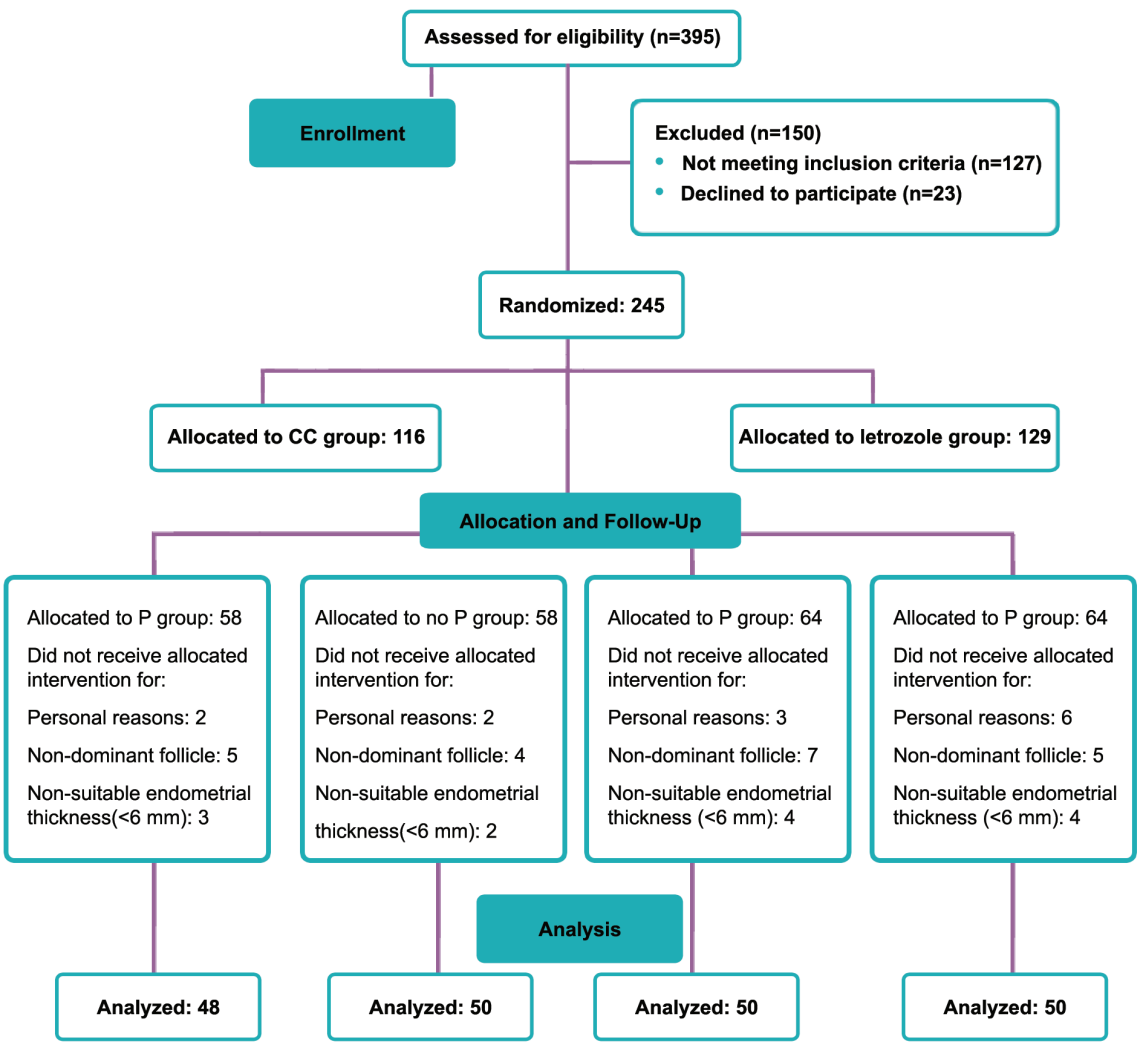
Patients flow chart.

Patients were eligible if they met following criteria: being 20-35 years of age; being married; not having non-classical adrenal hyperplasia, thyroid disorders and hyperprolactinemia; being Iranian; having effective speaking or listening skills; not having male factor for infertility; having normal uterine cavity and patency of fallopian tube as demonstrated by either hysterosalpingography (HSG) or diagnostic laparoscopy and hysteroscopy; and having Rotterdam diagnostic criteria. Based on random allocation sequence generated by one of researchers, enrolled participants (n=198) were divided into two main groups as follows: i. CC group (n=98) used a combination consisting of CC and HMG and ii. letrozole group (n=100) used a combination consisting of letrozole and HMG (Fig 1).

### Ovarian stimulation and luteal supplementation

On day 3 of the treatment cycle, baseline transvaginal ultrasounds scan (AU 350, Esaote, Milano, Italy) was performed. One physician carried out all sonograms and treatment protocols. The endometrial stripe was measured at its maximum anteroposterior thickness along the sagital axis of the uterine body. When there was no ovarian cyst on the scan, CC group received orally 100 mg clomiphene citrate (CC; Iran Hormone,Tehran, Iran) for 5 days starting on day 3 of the menstrual cycle, while letrozole group received 5 mg/day of letrozole (Femara; Novartis Pharma AG, Switzerland) from day 3 to day 7 of the menstrual cycle. It is noted that HMG contains follicle-stimulating hormone (FSH) and LH. The dose and duration of HMG treatment was adjusted according to the patient's response, after monitoring the follicular development including the number of growing follicles. Therefore, in both groups, at least 5 ampoules (Merional, IBSA, Switzerland) in total dosage of 150 IU containing FSH were applied intramuscularly (IM) daily from day 5 to day 10. After day 10 of the menstrual cycle, all patients were evaluated every other day by a transvaginal ultrasound. When one or more dominant follicle(s) reached .18 mm, ovulation was triggered in form of an IM injection of 5000 IU human chorionic gonadotropin (hCG) (Choriomon, IBSA, Switzerland). Afterward, all patients (n=245) were randomly divided into two sub-groups. The patients (n=122) who used P suppositories (Cyclogest, 400 mg vaginally; Alpharma, England) were included in LPS group, while the rest (n=123) who did not use the supplement were included in non-P cycles group. LPS group used P suppositories daily starting on the day after hCG and was continued for 14 consecutive days.

Outcome measure was the sign of chemical pregnancy (positive β-hCG test i.e. >25 IU/mL). Pregnancy testing was performed by determining the quantitative serum βhCG level on day 14 after P administration.

### Statistical analysis

For an expected pregnancy rate of 21% for patients with LPS and 12% for patients without LPS, a sample size of 50 patients per groups was required for a statistical power of 90% at a p level of 0.05. Socio-demographic characteristics of the groups were expressed as mean ± SD or case (percentage) elsewhere, while the collected data were compared using one-way analysis of variance (One-Way ANOVA) and Chi-squared tests. Comparison of chemical pregnancy between groups was performed by chi-squared test. Multivariable logistic regression was specified to evaluate association between pregnancy rate after LPS and variables of interest. The Statistical Package for Social Sciences (SPSS; SPSS Inc., Chicago, IL, USA) version 11.5 was used to assess the study data. P values were set as 0.05 for all analyses.

### Ethical considerations

The Ethics Committee of the Kashan Medical University approved the study. The protocol was explained to the patient's before they entered the study, while an informed consent was obtained from all.

## Results

We included 198 participants in the present study, who had completed treatment and follow up. The socio-demographic characteristics between groups were compared according to age, duration of infertility, endometrial thickness and number of dominant follicle. Results showed that there were no significant differences between the groups except for the age (Table 1).

**Table 1:** Socio-demographic characteristics of participants in CC and letrozole groups with and without using P


Variable	CC (N=98)		Letrozole (N=100)	
	Using P	Not using P	Using P	Not using P

**Age (Y) ^a^**	28.43(4.43)	25(3.50)	26.93(4.72)	25.8(3.30)
**Duration of infertility (month)**	46.47(38.01)	28.9(32.5)	37.7(35.27)	34.9(29.9)
**Total HMG (FSH received) ampoule**	5.81(0.56)	5.79(0.71)	5.78(0.59)	5.80(0.57)
**Endometrial thickness on hCG day(mm)**	7.62(0.88)	7.73(1)	7.76(1.09)	7.52(0.92)
**Number of follicle ≥18mm on hCG day**	1.77(1.27)	1.78(1.14)	1.78(1.09)	1.86(1.34)
**Pregnancy rate**	11(27.5)	7(17.5)	14(35)	8(20)


HMG; Human menopausal gonadotropin, FSH; Follicle-stimulating hormone, hCG; Human chorionic gonadotropin, CC; Clomiphene citrate, P; Progesterone and a; Values are mean (SD) or case (percentage). No significant differences between groups except for the age (p<0.001).

Progesterone supplementation was resulted in 10% higher pregnancy rate in LPS group than in non-P cycles group, although this difference did not reach statistical significance (p=0.08). LPS improved pregnancy rate in both CC (11 vs. 7, p=0.30) and letrozole (14 vs. 8, p=0.10) groups, although the difference is not significant. In addition, patients who used letrozole for ovulation induction had higher pregnancy rates when using intravaginal P support than CC group (14 vs. 11, p=0.40), although the difference is not significant.

To conduct thorough analysis on effect of P supplementation on the pregnancy rate with consideration of other confounders, we applied logistic regression. The effect of the parameters (Table 1) on pregnancy achievement after P supplementation was examined using a robust logistic regression model. Variables entered the model were selected by means of univariate comparisons between two group of patients who did or who did not achieve a pregnancy if p<0.05 (presence of pregnancy following P supplementation). The association between P supplementation and achievement of pregnancy was marginally significant (OR: 0.741, 95% CI: 0.539–1.019, p=0.06, Table 2).

**Table 2:** Logistic regression used as a dependent variable for the achievement of pregnancy


Dependent variable: incidence of pregnancy	P value	SE	Odds ratio	95% CI	
				Lower	Upper

**Independent variable**					
**Luteal support**	0.06	0.162	0.741	0.539	1.019
**Hosmer-Lemeshow test, p = 0.98.**					


SE; Standard error and CI; Confidence interval.

## Discussion

Currently, no information is available regarding the effect of LPS using P supplementation on pregnancy rate in stimulated cycles with combination therapies. This is the first study in which the effect of LPS with P on pregnancy rate was evaluated in Iranian women with PCOS who were treated with either CC or letrozole in combination with HMG for ovulation induction. Progesterone supplementation seemed to be of benefit in both the CC and letrozole treatment groups. The women with PCOS may inherently benefit from P supplementation in the luteal phase regardless of which medication is used for ovulation induction. P administration in PCOS patients has been demonstrated to decrease LH pulse frequency (11). In addition, the granulosa cells in women with PCOS may have intrinsic abnormalities in the response to both gonadotropin action and steroidogenesis. Granulosa cells in patients with PCOS have been verified to have some changes in response to LH before ovulation (12).

While development of several follicles to attain ovulation and large amounts of P are the main cause of the ovarian stimulation, the treatment overrides the physiological feedback mechanisms. The luteal phase of these cycles is characterized by a momentary high level of one or both hormones, which suppress the levels of LH and FSH (13). It has been recommended that the low level of LH may lead to lack of luteotrophic support determined by low P levels or short luteal phase (14). This is in agreement with previous studies by Erdem et al., and Maher et al., who found LPS with vaginal P positively affects the success of stimulated IUI cycles (5, 6), but other studies reported no benefit of LPS with either P, gonadotropin-releasing hormone (GnRH) agonist or hCG in patients who underwent ovulation induction (7, 8, 15).

In Balasch colleagues’ study (9), twenty infertile patients being treated with CC and hCG for induction of ovulation with a defective luteal phase were assigned into two groups of treatment using P supplementation or control. Their findings showed that success rates were similar in both groups (20 and 30%, respectively). It has been concluded that progestational agents have low therapeutic value in luteal phase deficiency induced by CC. It should be noted that oral and intravaginal P has been used in Balasch’s and present studies, respectively. P is usually well tolerated and the side effects encountered typically depend on the route of administration. But, the intravaginal method of P has gained popularity as a first option in luteal support treatment that is mainly due to patient comfort and practice efficiency (16). Local bioavailability in the uterus is greater after vaginal administration than with other routes (17) and this might be expected to result in an increased possibility of pregnancy.

It has been proposed that in IVF cycles, the use of GnRH analogs and gonadotropins cause multifollicular development, change of the hormonal environment, an increase in steroid serum concentration and an increase in risk of LPD (18). Nevertheless, in our study, the mean numbers of dominant follicles were 1.6. Some studies have noted that in cycles with mildly stimulated ovaries and less obvious follicular development (such as our study), there is no biological evidence to indicate that treatment with P in the luteal phase is necessary or improves pregnancy rates (19).

Tavanitou et al. (20) discovered that LH serum concentrations were significantly higher in patients administered CC. In other investigation have been shown that controlled ovarian stimulation with HMG in the follicular phase was an effective treatment for LPD associated with recurrent pregnancy loss (21). Understanding from induction ovulation with gonadotropins in hypophysectomized women had verified that it was essential to provide continued support in the form of hCG at least until the mid-late luteal phase (22). However, women undergoing ovarian stimulation are not totally hypogonadotrophic, so they need no support in luteal phase. Moreover, the half-life of hCG is relatively long if at least 5000 IU (dosage of hCG in our study) are used for ovulation induction, so a biologically significant amount persists for at least 10 days until the embryo starts secreting hCG (23).

In addition, in present study, patients who used letrozole for ovulation induction had higher pregnancy rates when using P as compared to CC group. Studies on effect of P supplementation in patients with PCOS using either CC or letrozole are limited. In a study by Montville et al. (10), they have shown that women with PCOS who used letrozole for ovulation induction had higher pregnancy rate when using intravaginal P support than CC group. Nevertheless, in this study, we did not compare pregnancy rate between P and control groups regardless taking medication. Aromatase inhibitors such as letrozole are hypothesized to maintain normal hypothalamic pituitary feedback mechanisms, and in case of ovulation induction in women with PCOS, may act to increase follicular sensitivity to FSH through increasing intrafollicular androgen levels. Unlike CC, letrozole does not antagonize the estrogen receptor in the endometrioum (24, 25). The lack of antagonism may contribute to increase pregnancy rate. In addition, the present study suggests that combination therapy of letrozole and luteal phase P improve pregnancy rate compared with letrozole alone. Letrozole may act to increase midluteal P levels after ovulation.

These observations would help to explain the benefits of CC, letrozole and HMG on the luteal phase that showed no significant relation in present study. Whether non-significant result is due to lack of LPD in mildly stimulated cycles (Table 1) or is due to the direct positive effect of CC and/or hMG or hCG on luteal phase is not clear.

Strengths of this study included matching properties such as duration of infertility, endometrial thickness and number of dominant follicles, indicating these confounders did not play a role in results. In addition, our trial included the same physician using the same clinical protocols for all patients. Most importantly, all patients followed the same lab protocols. We had attempted to adjust the results for parameters that were significantly different between the two study groups, but our conclusions are influenced by some limitation.

The obvious weakness is small sample size that did not provide an adequately powered analysis for the important confounders, so the tested outcome could be affected (pregnancy rate). Moreover, the lack of statistical significance of difference between groups in present study may be a result of not having the number of cycles required to reach appropriate statistical power. Perhaps the failure to observe a significant effect of P on pregnancy rate in the different studies may be explained in part by either small study sizes, inadequate statistical power to detect a significant difference, the use of different drugs for ovarian stimulation, as well as different types and dosages of P for LPS. Despite these limitations, our findings were the subject of thorough statistical analysis that added strength to our conclusions.

Undoubtedly there is a need for further prospective randomized studies, with larger samples and longer periods of follow-up, to confirm the real clinical benefit of luteal phase P administration (if any) before it is introduced into daily clinical practice.

## Conclusion

Our results suggest that LPS with P may improve pregnancy rate in PCOS patients treated with either CC or letrozole in combination with HMG.
